# Autistic traits modulate the influence of face masks on gaze perception

**DOI:** 10.1038/s41598-023-41900-0

**Published:** 2023-09-10

**Authors:** Elin H. Williams, Nicholas M. Thompson, Gareth McCray, Bhismadev Chakrabarti

**Affiliations:** 1 Centre for Autism, School of Psychology and Clinical Language Sciences, University of Reading, Reading, RG6 6DZ UK; 2 Faculty of Health, Education and Society, University of Northampton, Northampton, UK; 3 School of Medicine, Keele University, Keele, UK; 4 India Autism Centre, Kolkata, India; 5 Department of Psychology, Ashoka University, Sonipat, India

**Keywords:** Human behaviour, Social behaviour

## Abstract

Detecting when others are looking at us is a crucial social skill. Accordingly, a range of gaze angles is perceived as self-directed; this is termed the “cone of direct gaze” (CoDG). Multiple cues, such as nose and head orientation, are integrated during gaze perception. Thus, occluding the lower portion of the face, such as with face masks during the COVID-19 pandemic, may influence how gaze is perceived. Individual differences in the prioritisation of eye-region and non-eye-region cues may modulate the influence of face masks on gaze perception. Autistic individuals, who may be more reliant on non-eye-region directional cues during gaze perception, might be differentially affected by face masks. In the present study, we compared the CoDG when viewing masked and unmasked faces (N = 157) and measured self-reported autistic traits. The CoDG was wider for masked compared to unmasked faces, suggesting that reduced reliability of lower face cues increases the range of gaze angles perceived as self-directed. Additionally, autistic traits positively predicted the magnitude of CoDG difference between masked and unmasked faces. This study provides crucial insights into the effect of face masks on gaze perception, and how they may affect autistic individuals to a greater extent.

## Introduction

Eye gaze is a salient social cue. It reveals the focus of another individual’s attention, and, importantly, whether or not *we* are the focus of their attention. Thus, successful navigation of our social world depends on our ability to efficiently discern the focus of others’ gaze. Given its important role, it is perhaps not surprising that humans can detect subtle differences in gaze direction^1,2^ and are sensitive to detecting direct gaze from an early age^3^. This efficient detection of direct gaze plays an important role in the ability to understand the actions and intentions of others^4^.

While the ability to detect direct gaze is well refined, a liberal range of gaze angles is typically perceived as being self-directed (e.g.^5–7^). The range of gaze angles that one perceives as being self-directed is termed the “cone of direct gaze” (CoDG^5^). The width of the CoDG is approximately 9.55° in horizontal diameter (e.g.^5,8^), which broadly corresponds with the width of the human face^9^. Under more ambiguous conditions (e.g. gaze viewed from afar or in the dark) the width of the CoDG may be wider. Thus, when observers are less certain regarding where another individual is looking, they adopt a more liberal criterion and tend to overestimate the range of gaze angles perceived as being directed towards themselves^10–12^. Indeed, Balsdon and Clifford^8^ found that observers were more likely to judge gaze as direct in stimuli with increasing levels of eye-region noise (i.e. reduced reliability); faces wearing transparent sunglasses were least likely to lead observers to overestimate the range of gaze angles perceived as being directed towards them (i.e. a direct gaze bias), while opaque sunglasses were most likely to lead to a direct gaze bias. This overestimation of direct gaze under conditions of uncertainty minimises the risk of missing potential social interactions or failing to identify possible social threats. These are both, arguably, higher costs to pay than mistakenly perceiving averted gaze as direct^7^.

Although an individual’s CoDG width is a relatively stable trait^13^, there is considerable between-participant variability in the range of gaze angles perceived as self-directed (e.g.^6,14–16^). Certain characteristics may make observers more likely than others to perceive gaze as direct. For instance, individuals with high levels of social anxiety tend to exhibit a wider CoDG than those with lower levels of social anxiety^6,17,18^. In contrast, individuals with more autistic traits possess a narrower CoDG, suggesting that they are less likely than those with fewer autistic traits to overestimate direct gaze^19^. However, other studies report conflicting results; for example, Pell and colleagues^20^ found that individuals with Autism Spectrum Conditions (ASC) were as likely as non-autistic individuals to overestimate the perception of direct gaze when eye-region information was unreliable (i.e. when noise was added to the eye-region of faces).

Accurate gaze judgements are not based solely on the eye region, and can necessitate the integration of various directional cues, such as the angle of the nose^7,21^, and the direction in which the head^22–25^ or body^26^ is pointing. Misalignment between these different directional cues causes increased gaze uncertainty, resulting in an overestimation of direct gaze in averted compared to direct-facing heads^11,25^.

The relevance of different cues (e.g. eye orientation, head orientation, nose angle, or body orientation) during gaze perception, and their relative weightings, varies depending on the viewing condition^5^, context^8^, frame of reference^27^, and the cue’s reliability^28^. For instance, reduced reliability of, and consequently, increased observer uncertainty in, one cue (e.g. the eyes) likely reduces its relative weighting while increasing the weighting of another cue (e.g. the head)^5^. Perrett and colleagues^28^ suggest that while directional cues from the eyes are weighted heavily as a default, increased weighting is assigned to head orientation when information from the eyes is unreliable (e.g. when viewed from afar). Thus, under conditions where the reliability of eye-region cues is reduced, observers (1) exhibit a wider CoDG, and (2) rely more heavily on other available directional cues.

There is also evidence of individual differences in the relative weighting assigned to different directional cues. While non-autistic individuals weight eye-region cues more heavily than head orientation cues, autistic individuals may weight directional information from the body and head more than the eye-region^29,30^. Consistent with such findings, autistic individuals tend to exhibit a reduced propensity to fixate on the eye-region (e.g.^31–33^) and an increased propensity to fixate on the mouth-region in certain contexts (e.g.^31^). This pattern of decreased attention to the eye-region could be explained by the gaze aversion or gaze indifference hypotheses. Individuals exhibiting gaze aversion may purposefully avoid looking at others’ eyes because they find them aversive^34–37^. In contrast, individuals exhibiting gaze indifference may look less towards others’ eyes because they are not sensitive to the social significance of gaze^38–40^.

As multiple cues are integrated during gaze perception, it is possible that a reduction in the reliability of any cue, not just the eye-region, may influence the way in which gaze is perceived. While several studies have examined how eye-region reliability influences gaze perception (e.g.^8,10^), little is known about how a reduction in the reliability of other directional cues affects the perception of gaze. This is particularly relevant to investigate in the autistic population as autistic observers may rely less on eye-region cues for gaze perception, and thus might be disproportionately affected by a reduction in the reliability of other directional cues.

In many countries, the use of face masks was mandated during the COVID-19 pandemic in order to limit the spread of the virus among the population. As they occlude a large portion of the face, these masks could significantly affect social perception and interaction. Indeed, face masks impair the ability to recognise familiar and unfamiliar faces^41,42^, emotional facial expressions (^43,44^ though see^45^), and the intelligibility of speech^46^.

A recent study found that face masks do not alter gaze cueing of attention^47^; however, it is possible that the precision with which gaze is perceived may be reduced when directional cues from the lower face (e.g. nose angle) are occluded. This would manifest as increased gaze ambiguity and, consequently, an overestimation of direct-gaze (i.e. an increase in the width of the CoDG). Indeed, two recent studies, published subsequent to completion of data collection for the present study, reported an increase in the width of the CoDG when perceiving gaze in masked compared to unmasked faces^48,49^. Additionally, the relative extent to which people weight eye-region and non-eye-region cues during gaze perception could affect the extent to which face masks impact their perception of gaze direction. For example, in line with the gaze aversion hypothesis^34–37^, autistic individuals, who may rely more heavily on non-eye-region directional cues (e.g. nose angle or head orientation) during gaze perception, might not shift their gaze to the eye-region when non-eye-region cues are occluded. Consequently, they may experience increased gaze ambiguity, and thus a larger increase in CoDG width. Alternatively, in line with the gaze indifference hypothesis^38–40^, the presence of face masks could induce autistic individuals to attend more to the, arguably, more informative eye-region cues. Such an effect might reduce the influence of other directional cues, resulting in more sensitive perception of direct gaze, and thus a narrower CoDG.

Few studies have examined how face masks, which occlude the lower portion of the face, influence gaze perception. Further, individual differences in the impact of face masks on gaze perception is unclear. Accordingly, this pre-registered study investigated whether, and how, a reduction in the reliability of lower face cues affects gaze perception. Participants viewed both masked and unmasked faces that were either facing directly toward them (i.e. direct head orientation) or averted away from them (i.e. averted head orientation). The orientation of the eyes of the faces were also manipulated such that they varied from looking 15° to the left of the observer to looking 15° to the right of the observer, generating 13 unique eye orientations (i.e. − 15°, − 12°, − 9°, − 6°, − 3°, − 1°, 0°, 1°, 3°, 6°, 9°, 12°, 15°). Participants were asked to judge whether or not faces were looking at them in a two-alternative forced choice (2AFC) task. We measured the width of the CoDG for each participant, while also examining whether participant-reported autistic traits modulate the relationship between face masks and gaze perception.

## Results

**Figure 1:**
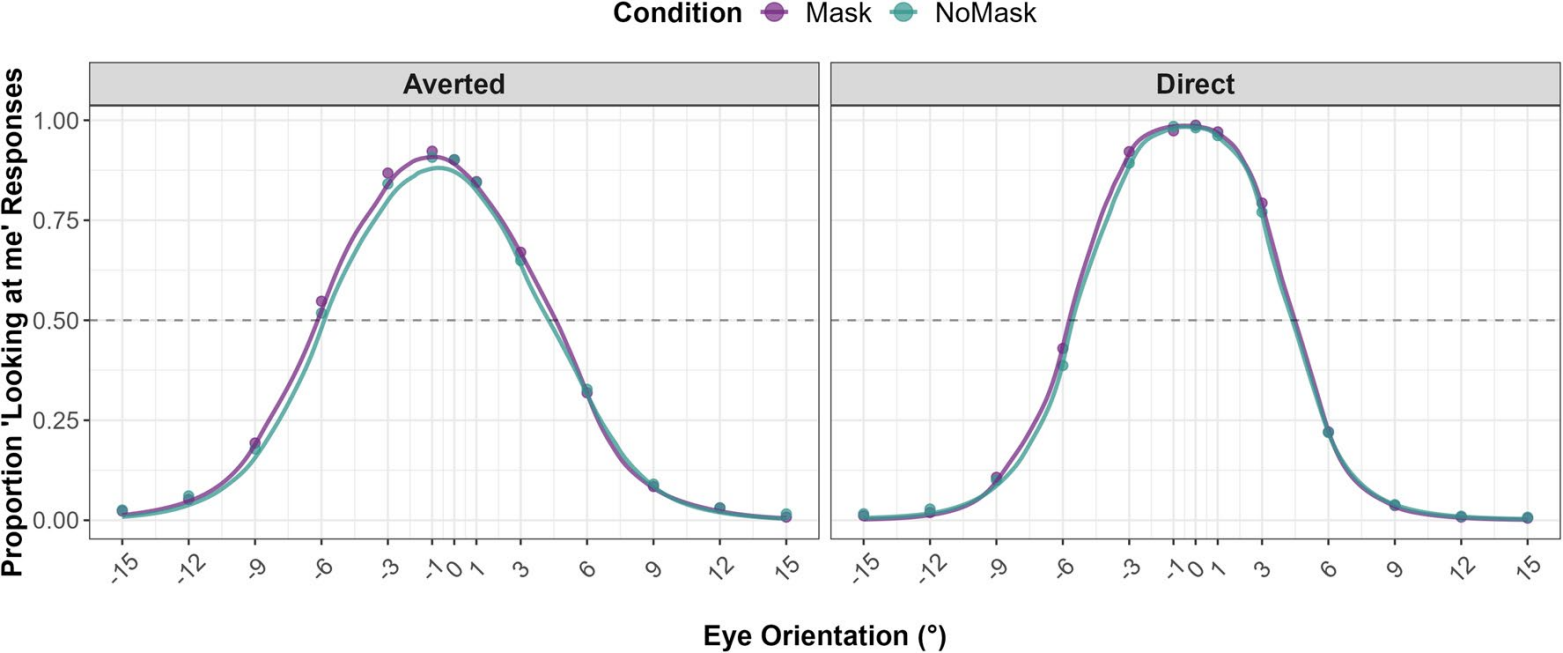
Responses to the 2AFC task. The filled points show the actual proportion of responses, while the solid lines represent the fitted data. The horizontal dashed line represents the point of subjective equality (PSE). Data are averaged over all participants for illustration purposes.

**Figure 2:**
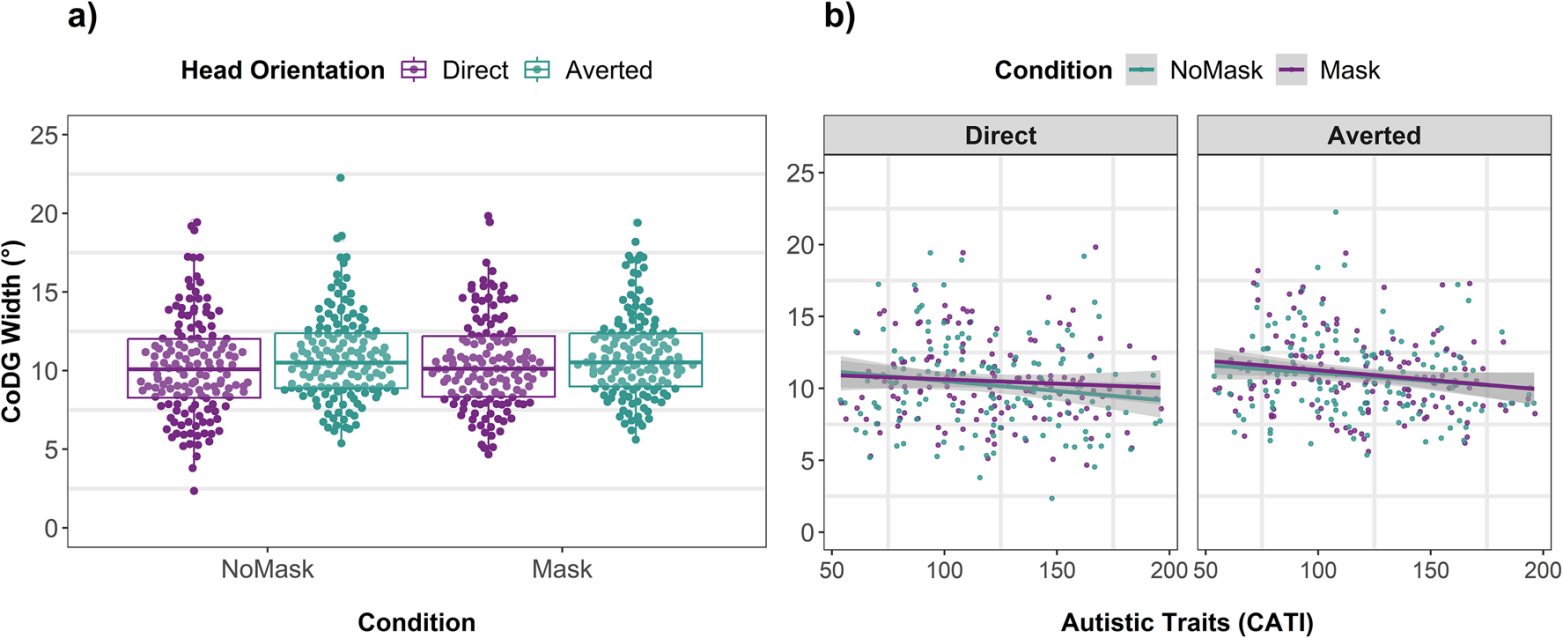
(**a**) Individual CoDG widths plotted as a function of *Mask Condition* and *Head Orientation*, jittered proportionally to the density. Boxplots represent 25th and 75th percentiles, while whiskers represent upper and lower values within 1.5*interquartile range. (**b**) Each participant’s CoDG width is plotted as a function of the *Mask Condition*, *Head Orientation*, and the participant’s self-reported autistic traits measured by the Comprehensive Autistic Trait Inventory (CATI^50^).

Figure 1 shows the group-averaged responses to the 2AFC task. The peaks of the curves represent the eye orientation, in degrees, at which participants mostly perceive direct gaze across *Mask Conditions* and *Head Orientations*. The results of the linear mixed-effects model (Table 1, Fig. 2) revealed that the CoDG was 0.3° wider in the masked condition (estimated marginal mean (EMM) = 11°; SE = 0.23; CI [10.6, 11.5]) compared to the unmasked condition (EMM = 10.7°; SE = 0.23; CI [10.3, 11.2]) (β =  − 0.16; SE = 0.06, *t* (416.92) =  − 2.52; *p* = 0.012; CI [− 0.29, − 0.04]). The CoDG was 0.7° wider for averted heads (EMM = 11.2°; SE = 0.23; CI [10.8, 11.7]) compared to direct-facing heads (EMM = 10.5°; SE = 0.23; CI [10.1, 11.0]) (β =  − 0.34; SE = 0.06, *t* (416.94) =  − 5.32; *p* < 0.001; CI [− 0.46, − 0.22]). No significant interaction between *Mask Condition* and *Head Orientation* was observed (Fig. 2a). We found a significant effect of *Sex* (β =  − 0.56; SE = 0.23, *t* (148.76) =  − 2.49; *p* = 0.013; CI [− 1.01, − 0.12]), where the CoDG for males was 1.1° wider (EMM = 11.4°; SE = 0.36; CI [10.73, 12.2]) than for females (EMM = 10.3°; SE = 0.26; CI [9.81, 10.80]). Autistic traits did not significantly predict the width of the CoDG, nor did they interact with *Head Orientation* or *Mask Condition* (Fig. 2b).

**Table 1:** Linear mixed-effects model summary.

Predictors	CoDG width (°)			
	Estimates	95% CI	t statistic	*p*
Mask condition (MC)	− **0**.**16**	− **0**.**29** to − **0**.**04**	− **2**.**52**	**0**.**012**
Autistic traits (CATI)	− 0.22	− 0.63 to 0.20	− 1.03	0.302
Head orientation (HO)	− **0**.**34**	− **0**.**47** to − **0**.**21**	− **5**.**32**	** < 0**.**001**
Age	0.36	− 0.07 to 0.78	1.66	0.097
Sex	− **0**.**56**	− **1**.**01** to − **0**.**12**	− **2**.**48**	**0**.**013**
MC * CATI	− 0.09	− 0.21 to 0.04	− 1.36	0.175
MC * HO	− 0.01	− 0.14 to 0.11	− 0.18	0.859
CATI * HO	0.02	− 0.11 to 0.14	0.27	0.784
MC * CATI * HO	− 0.08	− 0.21 to 0.05	− 1.24	0.215
Random effects				
σ^2^	2.30			
τ_00_ _PID_	6.01			
ICC	0.72			
N_PID_	157			
Observations	578			
Marginal R^2^/conditional R^2^	0.086/0.747			

Bolded font indicates *p* values less than 0.05.

### Exploratory analysis

Contrary to expectations, we found no evidence to suggest that autistic traits influence the width of the CoDG or that they interact with *Mask Condition*. As noted in the *Introduction,* it is possible that some autistic individuals who exhibit gaze aversion^34–37^ place greater reliance on non-eye-region cues during gaze perception and, therefore, when these cues are occluded, may experience an increase in gaze ambiguity (i.e. widening of CoDG). However, other autistic individuals who exhibit gaze indifference^38–40^, may shift their attention to attend to the eye-region when other cues are unreliable, which may lead to more sensitive perception of gaze (i.e. narrowing of CoDG). If these contrasting effects were both present within our data, it might preclude the ability to detect an effect of autistic traits in our model.

To investigate this possibility, we conducted an exploratory analysis wherein we computed a composite ‘*Mask Effect*’ score for each participant, for both direct and averted heads (n.b. this was not pre-registered). This *Mask Effect* was calculated by subtracting the width of the CoDG in the unmasked condition from the masked condition. As we hypothesised that participants reporting more autistic traits may show either a greater widening or a greater narrowing of the CoDG, we converted *Mask Effect* scores into absolute values in order to characterise the overall effect of face masks on gaze perception, regardless of the direction of this effect.

We fit linear mixed-effects models using restricted maximum-likelihood to investigate whether *Mask Effect* is predicted by autistic traits, *Head Orientation*, and their interaction. Participants were entered as random effects and sex and age were entered as fixed-effect covariates (*formula: Mask Effect* ~ Autistic Traits * Head Orientation + *Age* + *Sex* + *(1 | Participant)*). Twelve influential observations (3.9%) were excluded based on the criterion Cook’s D greater than 4 times the group average Cook’s D (> 0.10).

**Figure 3:**
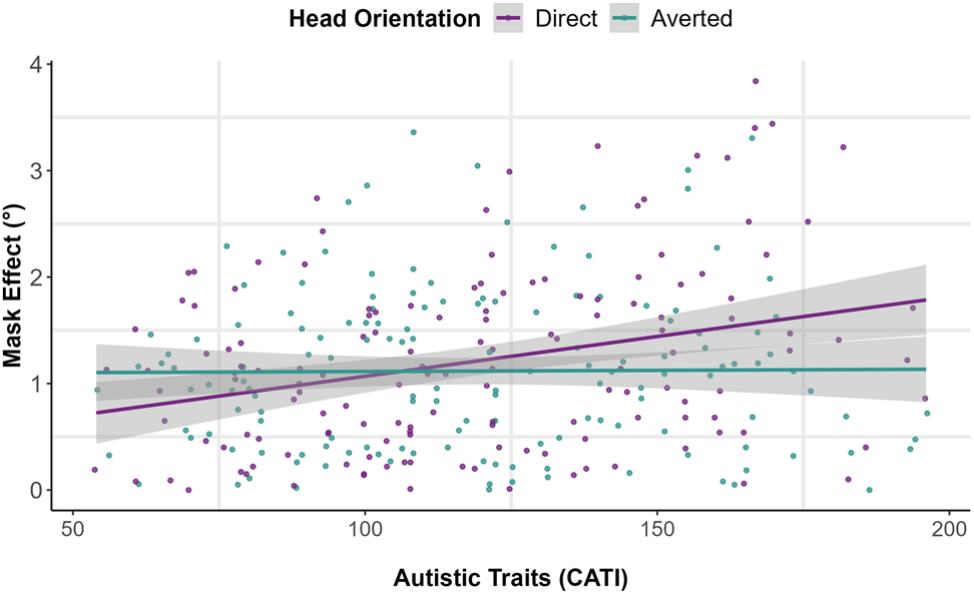
Each participant’s absolute *Mask Effect* score, representing the overall effect of face masks, is plotted as a function of *Head Orientation* and the participant’s self-reported autistic traits as measured by the CATI. The shaded grey areas represent the standard error of the estimates.

The results (Table 2, Fig. 3) revealed that *Mask Effect* was significantly predicted by autistic traits (β = 0.14; SE = 0.05, *t* (141.87) = 2.87; *p* = 0.005; CI [0.05, 0.24]) such that individuals reporting more autistic traits were more affected by the presence of a face mask (i.e. the magnitude of the *Mask Effect* was greater for those reporting more compared to fewer autistic traits). Additionally, a significant interaction was observed between autistic traits and *Head Orientation* (β = 0.12; SE = 0.05, *t* (142.81) = 2.75; *p* = 0.007; CI [0.04, 0.21]). We conducted a simple slopes analysis to investigate this two-way interaction; this revealed that the slope of autistic traits was significantly different from zero in the direct head condition (β = 0.27, SE = 0.07, t = 3.99, *p* < 0.001) but not in the averted head condition (β = 0.02, SE = 0.07, t = 0.25, *p* = 0.80).

**Table 2:** Linear mixed-effects model summary for exploratory analysis with composite *Mask Effect* score.

Predictors	Mask effect (°)			
	Estimates	95% CI	t statistic	*p*
Autistic traits (CATI)	**0**.**14**	**0**.**04** to **0**.**24**	**2**.**87**	**0**.**004**
Head orientation (HO)	0.04	− 0.04 to 0.13	1.00	0.319
Age	0.00	− 0.01 to 0.01	0.21	0.834
Sex	− 0.05	− 0.15 to 0.06	− 0.85	0.399
CATI * HO	**0**.**12**	**0**.**04** to **0**.**21**	**2**.**75**	**0**.**006**
Random effects				
σ^2^	0.59			
τ_00_ _PID_	0.05			
ICC	0.08			
N_PID_	156			
Observations	292			
Marginal R^2^/conditional R^2^	0.056/0.129			

Bolded font indicates *p* values less than 0.05.

## Discussion

This study examined how wearing a face mask impacts upon judgments of direct gaze in the observer. We found that the CoDG was wider for masked faces compared to unmasked faces, and that masks influenced direct gaze judgment to a greater extent in individuals with high autistic traits.

Although recent studies have investigated how face masks affect emotion perception^43,44^, person perception^41,42^, and speech intelligibility^46^, little is known about whether, and how, they affect gaze perception. Our results suggest that much like when eye-region cues are less reliable, a reduction in the reliability of lower face cues increases gaze direction uncertainty, thereby increasing the range of angles at which gaze is perceived as self-directed^10–12^. Two recent studies, published subsequent to completion of data collection for the present study, also reported an increase in the width of the CoDG when perceiving gaze in masked compared to unmasked faces^48,49^. While the width of the CoDG increased only slightly for masked faces in our study (0.3°), this is closely aligned with an increase of 0.28° reported by^48^. It is important to note that this pattern of increased CoDG was not observed uniformly across all participants. In the present study, 59% of participants exhibited a wider CoDG for masked faces compared to unmasked faces, while 41% of participants showed the opposite effect. This corresponds to the findings of^48^ who found that 61% of participants exhibited a wider CoDG for masked faces in comparison to unmasked faces. While the data from the present study do not enable an unequivocal explanation for this finding, as we go on to discuss, one possible explanation relates to individual differences between participants.

The CoDG was 0.7° wider when observers perceived gaze in averted heads compared to direct-facing heads. It is likely that misalignment between head and eye cues increases observer uncertainty when perceiving gaze, which could lead to a more liberal threshold for assuming that gaze is self-directed^10,11^. We did not find a significant interaction between *Mask Condition* and *Head Orientation*. This suggests that the presence of face masks does not lead to a greater increase in observer uncertainty in averted compared to direct-facing heads, nor does it improve observer accuracy in averted heads by redirecting attention to the eye-region of masked faces, thereby reducing any bias caused by a misaligned head orientation.

Interestingly, we found that sex significantly predicted the width of the CoDG in our sample, such that males had a wider CoDG than females. A number of other studies have also shown that males are more likely to overestimate direct gaze than females (e.g.^51,52^). Studies suggest that females are more accurate at perceiving gaze than males (e.g.^53,54^), which may be explained by their increased fixations towards the eyes of others^55^. Accordingly, it is possible that females are more sensitive to discriminating between averted and direct gaze, hence their narrower CoDG.

A further aim of this study was to examine whether autistic traits modulate the effect of face masks on the CoDG. Our primary analysis found no evidence to suggest that autistic traits predict the width of the CoDG, nor that they interact with *Mask Condition* or *Head Orientation*. However, as noted in the *Introduction*, some autistic individuals may rely more heavily on non-eye-region directional cues during gaze perception^29,30^. In the absence of these cues, participants who exhibit gaze aversion^34–37,56^ might be less likely to redirect their attention to the eye-region, which could result in increased gaze uncertainty and a wider CoDG. In contrast, autistic observers who typically attend less to the eye region because they do not find the eyes engaging or informative because of a gaze indifference^38–40^, rather than because they find them aversive, may redirect their attention to the eye-region, resulting in more precise gaze judgments and a narrower CoDG. The presence of both of these effects within our data would mean that under masked conditions, some participants with more autistic traits would show a narrowing of the CoDG, while others would show a widening of the CoDG. These opposing effects would thus cancel out the other and preclude the ability of our main analysis to detect any effect related to autistic traits.

To test this hypothesis, we conducted an exploratory analysis which examined the relationship between autistic traits and the overall impact of face masks on gaze perception (i.e. the magnitude of the difference between masked and unmasked conditions, independent of the direction of this difference). We found that participants who reported more autistic traits exhibited a larger mask effect compared to participants who reported fewer autistic traits (i.e. they showed a greater magnitude of widening or narrowing of the CoDG in the masked relative to the unmasked condition). This supports the suggestion that occluding the lower part of the face results in a widening of the CoDG for some autistic individuals and a narrowing of the CoDG for others. As noted above, one possible factor that could explain this difference is the extent to which an individual exhibits gaze aversion, which could relate to underlying social anxiety. While we captured a measure of social anxiety in this study, this was too highly correlated with our measure of autistic traits to enable an examination of the effect of social anxiety in those with high autistic traits. Future research should seek to recruit autistic individuals with and without comorbid social anxiety disorders, as well as identify other characteristics that could explain the differential effects of face masks on CoDG. In view of the extensive literature examining social anxiety, social reward processing, and gaze aversion/indifference in autistic populations, we would recommend these constructs as preliminary targets for future research.

Our exploratory analysis also demonstrated an interaction between autistic traits and head orientation, whereby the heightened mask effect in participants with more autistic traits was observed only for direct-facing heads. One possible explanation for the observed difference in this effect between direct-facing and averted heads relates to the gaze aversion and gaze indifference hypotheses. The effect of face masks may be magnified in individuals exhibiting gaze aversion or gaze indifference. Direct-facing heads may be perceived as more aversive than averted heads by individuals who exhibit gaze aversion; this may result in greater gaze uncertainty in the masked condition due to avoidance of the eye-region. Conversely, observers who exhibit gaze indifference may greatly benefit from an occlusion of lower-face cues in the masked condition. When lower-face cues are unreliable they may shift their attention to the eye-region and perceive gaze with greater sensitivity; this effect may be magnified in direct-facing heads as the bias from an averted head is reduced.

It is important to highlight certain limitations of the present study. Firstly, this study was conducted entirely online. While online research has seen a recent surge in popularity, it is not without its inherent limitations, such as reduced control over participants and the conditions in which studies are completed. That said, previous studies have successfully conducted similar experiments online (e.g.^57^) and recent work has demonstrated reasonable concordance between measures collected online and in a lab setting (e.g.^58,59^). Nonetheless, it would be beneficial for future work to replicate this study in a lab setting. Additionally, data were collected in the UK and USA between May and December 2022. COVID-19 restrictions, such as mandatory face masks in public spaces, had only recently been lifted in the UK at this time, while face masks were still mandatory in many US states. Country-specific mask requirements, and subsequent experience with masks, may have an effect on how participants from different countries perceive gaze in masked vs unmasked faces; examination of such effects was beyond the scope of the present study.

Secondly, in prioritising experimental control over ecological validity, only a static, computer-generated, white male face was presented to participants. While this enabled greater systematic control in generating various combinations of eye and head angles, we acknowledge that gaze perception in dynamic, real-life social interactions is far less predictable, and that it may differ depending on the sex of the face presented to participants (e.g.^52,60^).

Finally, although we enriched our sample by recruiting an additional group of participants with confirmed autism spectrum diagnoses, we did not set out to compare gaze perception between autistic and non-autistic individuals. Future work should seek to build on the present findings by examining the effect of masks on CoDG in autistic and non-autistic groups matched in terms of IQ, age, and sex. Additionally, it would be interesting for future work to include eye-tracking to examine differences in visual gaze patterns to masked and unmasked faces, both between and within these groups.

The results of this study indicate that, overall, the occlusion of lower face cues increases gaze uncertainty, which results in an overestimation of direct-gaze, similar to when eye-region cues are unreliable^8,10,11^. However, our findings suggest that individuals with more autistic traits are disproportionately affected by the presence of face masks, which manifests as a widening of the CoDG (i.e. more uncertainty) in some observers, and a narrowing of the CoDG (i.e. higher precision) in others. Understanding the factors that might explain why an autistic individual experiences a widening or a narrowing of the CoDG under these conditions is a key question to be addressed by future work in this area. As the use of face masks is expected to remain higher than pre-pandemic levels^61^, it is imperative that research examines the impact of such interventions on social interactions. This study provides crucial insights into how the impact of face masks on gaze perception may affect autistic individuals to a greater extent.

## Methods

### Participants

Participants were recruited online either via Amazon’s Mechanical Turk (MTurk) or the University of Reading SONA systems. In order to ensure an adequate spread of autistic traits, we enriched our sample by recruiting additional autistic participants from the University of Reading Centre for Autism adult participant database (n.b. this aspect of the study was not pre-registered. The results of an analysis excluding autistic participants are presented in Supplementary Materials 1). Participants who are registered on this database have a confirmed clinical autism spectrum diagnosis from a registered health professional. Participants were reimbursed for approximately 40 min of their time.

Studies investigating individual differences are more likely to find small effect sizes^62^. We conducted an a-priori power analysis for correlations in G*Power^63^ which showed that a sample of N = 120 participants can detect small-medium (0.25) correlations with 80% power (n.b. we pre-registered a sample of N = 120). Due to over-recruiting to account for data loss, and the additional recruitment of participants with ASC diagnoses, a total of N = 163 participants was tested in this study. This sample comprised n = 99 recruited from MTurk, n = 29 recruited from SONA, and n = 35 recruited through our autism database. Participants were only included for analysis if they obtained a total attention score of 75% or more (see details of attention checks in the “Procedure” section); n = 161 were included for analysis following this criterion. After applying the exclusion criteria detailed in the “Data analysis” section, n = 157 participants were retained for analysis (M*age* = 37.59, SD = 13.39, 103 females).

All participants provided written informed consent, and ethical approval was granted by the University of Reading, School of Psychology and Clinical Language Sciences Ethics Committee (ethical approval number: 2022-061-BC), which adheres to the ethical guidelines presented in the 6th (2008) Declaration of Helsinki.

### Stimuli

**Figure 4:**
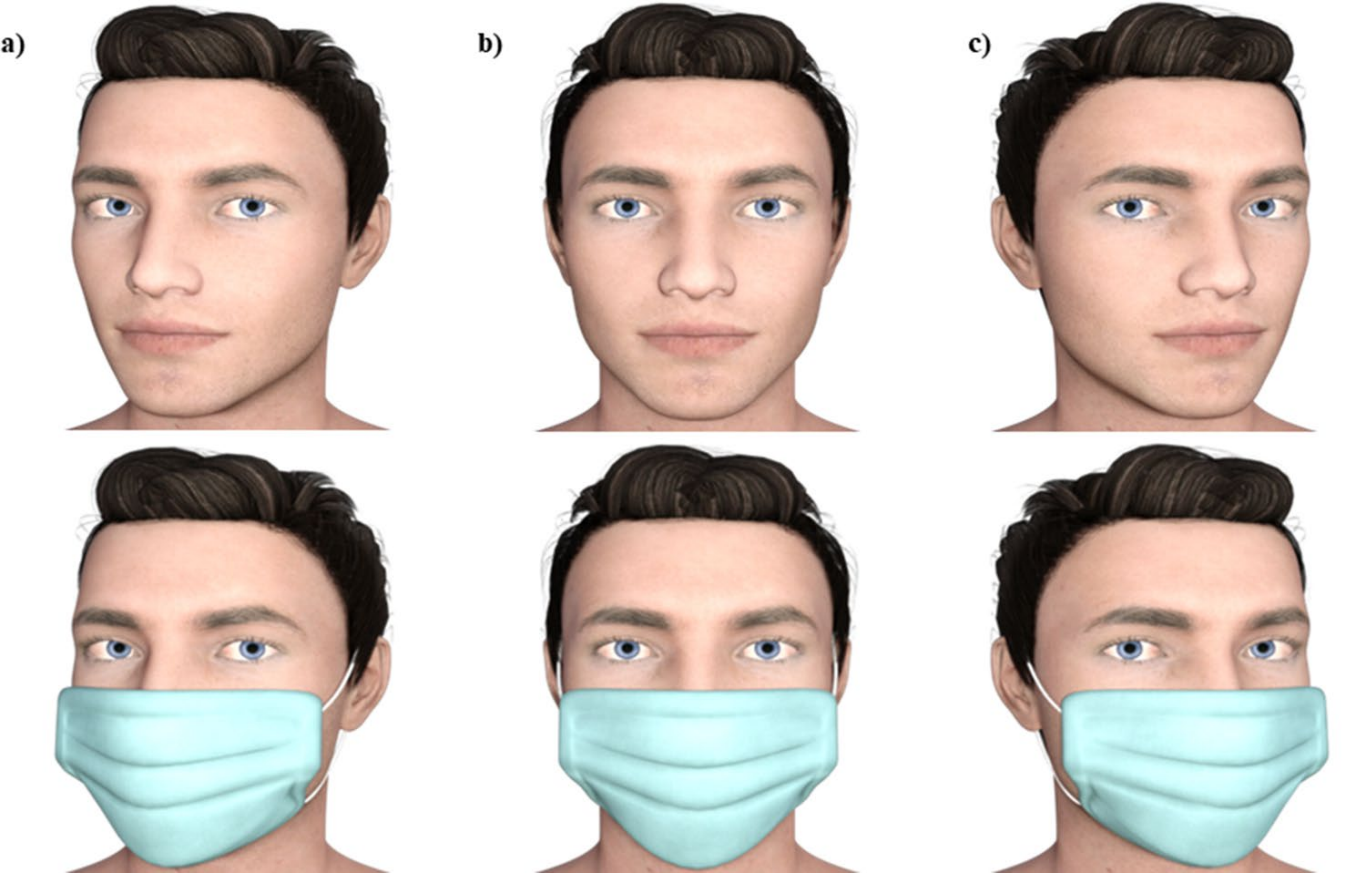
Example masked and unmasked faces with eyes oriented 0° and with heads (**a**) oriented − 15° to the left of an observer, (**b**) directly facing the observer (0°), or (**c**) oriented 15° to the right of an observer.

One male face stimulus was generated using Daz software (http://www.daz3d.com/). The orientation of the eyes of this stimulus were manipulated such that they varied from looking 15° degrees to the left of an observer to 15° to the right of an observer, generating 13 unique eye orientations (i.e. − 15°, − 12°, − 9°, − 6°, − 3°, − 1°, 0°, 1°, 3°, 6°, 9°, 12°, 15°). Additionally, the head orientation of the stimulus was manipulated to either directly face the observer (0°), or be turned to their left (− 15°) or right (15°) (Fig. 4). For the purpose of reducing the effect of stimulus asymmetry on gaze perception, the face was flipped along the vertical axis for half of the trials. A second set of stimuli was generated using Daz software by adding a surgical-type face mask to the first set.

### Procedure

**Figure 5:**
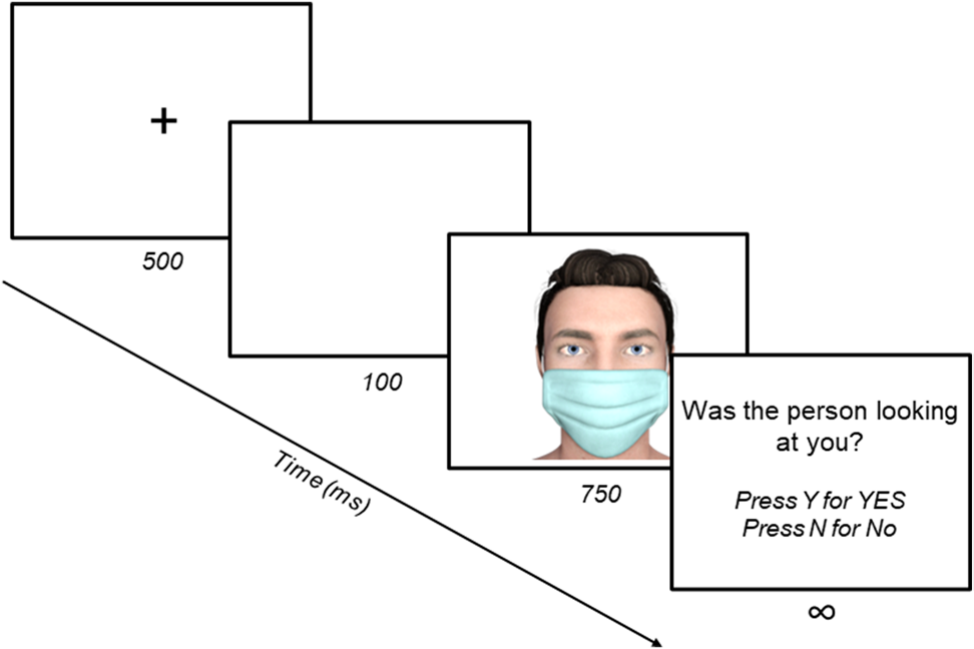
Example trial sequence. Each trial began with a central fixation cross presented for 500 ms. A blank screen then appeared for 100 ms, before the stimulus was displayed for 750 ms.

The experimental task (Fig. 5) was hosted on Gorilla Experiment Builder^64^, and participants were only able to complete the task from a laptop or desktop computer. Each trial began with a central fixation cross presented for 500 milliseconds (ms). A blank screen then appeared for 100 ms, before the stimulus was displayed (image size: 425 × 543 pixels) at full resolution for 750 ms. After the presentation of the stimulus, participants were asked to respond as to whether or not the face was looking at them. In a 2AFC task, participants used the ‘Y’ and ‘N’ letters on their keyboard to record ‘Yes’ and ‘No’ responses respectively (n.b. no explicit instructions were given regarding which fingers participants should use to respond). The next trial started after participants made a response.

With 8 repetitions of each combination of eye orientation (13 levels), head orientation (3 levels), and mask condition (2 levels), each participant completed 624 trials in total across 8 blocks. Masked and unmasked conditions were presented across separate blocks. Breaks could be taken in between blocks of trials. Participants firstly completed 6 practice trials to familiarise themselves with the task. The practice task displayed only trials for which the answer to the question ‘Was the person looking at you?’ was relatively clear (e.g. a head oriented 0° with eyes also oriented 0° should be a simple ‘Yes’ response, while a head turned − 15° with eyes also oriented − 15° should be a simple ‘No’ response).

As the task was completed online, attention checks were presented randomly throughout to ensure participants were adequately engaged. To reduce the likelihood of submission from bots or random responses from participants, we included free-text responses to simple questions (e.g. ‘Was the face wearing a face mask?’, ‘What is your age?’).

Participants completed the CATI as a measure of self-reported autistic traits. The mean of our full sample of participants (*M* = 119.53, *SD* = 34.65) is reflective of the mean typically found in a non-autistic sample^50^. English and colleagues^50^ suggested that a cut-off of 134 on the CATI best discriminates autistic from non-autistic participants. With the exception of two individuals, all participants recruited through our autism database scored above this threshold (*M* = 161.97, *SD* = 18.61). As an increased width of CoDG has been associated with social anxiety^6,17,18^, we also administered the Social Interaction Anxiety Scale (SIAS^65^). Peters^66^ defined the SIAS cut-off score for social anxiety as 36; our full sample of participants exhibited slightly elevated levels of social anxiety (*M* = 37.12, *SD* = 19.37). Both the CATI and SIAS questionnaires included two attention questions to reduce the likelihood of participants responding randomly to the questionnaire items.

### Data analysis

#### Cone of direct gaze calculation

The CoDG was calculated by fitting curves to the values for eye orientation, similar to the methodologies presented in previous studies of this nature (e.g.^14,24^). Specifically, a pair of logistic curves were fitted to model the probability of a participant considering gaze as self-directed given the eye orientation. One curve was fitted to eye orientations to the right of, and including, 0 degrees. A second curve was fitted to the eye orientations to the left of, and including, 0 degrees. The logistic functions had unique locations, but shared a common slope parameter, to aid in making the fitting more robust. The curves were fitted by minimising the sum of squared residuals for both functions concurrently. We extracted the 50% probabilities (i.e. the PSE) of considering gaze as direct for both the left and right gaze directions [the code is publicly available on our project OSF page (https://osf.io/gtjc2/?view_only=945076407acb49f68ca4139c58fecad3)]. The width of the CoDG was calculated as the sum of the absolute left and right-side PSE.

#### Analysis

Using the lmerTest package^67^ in R (version 4.1.2) we fit linear mixed-effects models using restricted maximum-likelihood to investigate whether the width of the CoDG is predicted by the presence of a face mask, autistic traits, head orientation, and their interaction. Participants were entered as random effects (*formula: CoDG* ~ Mask Condition * Autistic Traits * Head Orientation + *Age* + *Sex* + *(1 | Participant)*). Autistic traits and age were mean-centred and scaled. Data from the two averted head orientations (i.e. − 15° and 15°) were averaged in order to compare responses between averted and direct-facing heads. Due to high correlation between scores from the CATI and SIAS (*r* = 0.78, *p* < 0.001), SIAS could not be entered as a covariate in this model; results from a model including SIAS are presented in Supplementary Materials 2.

Thirty-eight influential observations (5.9%) were excluded from the model based on the criterion Cook’s D greater than 4 times the group average Cook’s D (> 0.12). Significance of fixed effects were determined using Satterthwaite approximations of degrees of freedom using the lmerTest package, limiting Type-1 errors but maintaining power^68^.

### Supplementary Information

Supplementary Information.

## Data Availability

The data and stimuli from this pre-registered (https://aspredicted.org/FMT_D8G) study are publicly available online (https://osf.io/gtjc2/?view_only=945076407acb49f68ca4139c58fecad3), and we report all data exclusions and measures obtained.
