# Supplementary material for: Autistic traits modulate the influence of face masks on gaze perception
Source: Sci Rep. 2023 Sep 10;13:14921. doi: 10.1038/s41598-023-41900-0 (PMC10493222; doi:10.1038/s41598-023-41900-0)
Supplement: Supplementary file 1 — Supplementary Information. [file 41598_2023_41900_MOESM1_ESM.pdf]

Autistic traits modulate the influence of face masks on gaze perception

Supplementary Materials

Elin H. Williams[1*], *Nicholas M. Thompson,*[1,2], *Gareth McCray*[3], *& Bhismadev Chakrabarti*[1,4,5]

[1]Centre for Autism, School of Psychology and Clinical Language Sciences, University of Reading, Reading, UK

[2]Faculty of Health, Education and Society, University of Northampton, Northampton, UK

[3]School of Medicine, Keele University, Keele, UK

[4]India Autism Centre, Kolkata, India

[5]Department of Psychology, Ashoka University, India

Supplementary Materials 1

Analysis

As the recruitment of additional participants with confirmed clinical autism spectrum diagnoses was not pre-registered, we reran the analyses while excluding participants with confirmed ASC diagnoses (Supplementary Table S1). Twenty-eight influential observations (6%) were excluded based on the criterion Cook's D greater than 4 times the average Cook's D (> 0.12).

Results

When excluding autistic participants, the width of the CoDG did not differ between masked and unmasked conditions (β = -0.14; SE = 0.08, t (329.48) = -1.78; p = 0.075; CI [-0.29, 0.01]). The width of the CoDG was 0.5° wider when participants saw averted heads (EMM = 11.1°; SE = 0.24; CI [10.6, 11.6]) compared to direct-facing heads (EMM = 10.6°; SE = 0.24; CI [10.1, 11.0]) (β = -0.19; SE = 0.08, t (329.55) = -2.46; p = 0.014; CI [-0.34, -0.04]). There was no significant interaction between Mask Condition and Head Orientation. We found a significant effect of sex (β = -0.54; SE = 0.25, t (119.01) = -2.19; p = 0.030; CI [-1.02, -0.06]), where the width of the CoDG for males was 1.1 ° wider (EMM = 11.4°; SE = 0.38; CI [10.60, 12.10]) than for females (EMM = 10.3°; SE = 0.28; CI [9.74, 10.80]).

Supplementary Table S1. Linear mixed-effects model summary excluding participants with confirmed ASC diagnoses

	CoDG width (°)			
Predictors	*Estimates*	*95% CI*	*t Statistic*	*p*
Mask Condition (MC)	-0.14	-0.29 – 0.01	-1.78	0.075
Autistic Traits (CATI)	-0.34	-0.87 – 0.20	-1.23	0.220
**Head Orientation (HO)**	**-0.19**	**-0.34 – -0.04**	**-2.46**	**0.014**
Age	0.03	-0.01 – 0.06	1.52	0.128
**Sex**	**-0.54**	**-1.02 – -0.06**	**-2.19**	**0.029**
MC * CATI	-0.07	-0.24 – 0.09	-0.86	0.389
MC * HO	-0.09	-0.24 – 0.06	-1.14	0.255
CATI * HO	0.16	-0.00 – 0.33	1.92	0.056
MC * CATI * HO	-0.16	-0.33 – 0.01	-1.86	0.064
**Random Effects**				
σ^2	2.24			
$\tau_{00\ PID}$	5.34			
ICC	0.70			
N_{PID}	124			
Observations	456			
Marginal R^2 / Conditional R^2	0.095 / 0.733			

Bolded font indicates p values less than 0.05

Supplementary Materials 2

Analysis

Due to high collinearity between autistic traits and social anxiety scores, the effect of social anxiety on the width of CoDG was analysed in a separate model. Using the lmerTest package (Kuznetsova et al., 2017) in R (version 4.1.2; R Core Team, 2021) we fit linear mixed-effects models using restricted maximum-likelihood to investigate whether the width of the CoDG is predicted by the presence of a face mask, social anxiety, head orientation, and their interaction. Participants were entered as random effects (*formula: CoDG ~ Mask Condition * Social Anxiety * Head Orientation + Age + Sex + (1 | Participant)*). Autistic traits and age were mean-centred and scaled. Thirty-eight influential observations (5.9%) were excluded from the model based on the criterion Cook's D greater than 4 times the average Cook's D (> 0.12).

Results

The results of the linear mixed-effects model, including social anxiety scores as a predictor (Supplementary Table S2), are similar to the results from the model including autistic traits as a predictor. The width of the CoDG was, on average, 0.3° wider in the masked condition (EMM = 11°; SE = 0.24; CI [10.6, 11.5]) compared to the unmasked condition (EMM = 10.7°; SE = 0.24; CI [10.2, 11.1]) (β = -0.18; SE = 0.07, t (378.04) = -2.75; $p = 0.006$; CI [-0.31, -0.05]). The width of the CoDG was also 0.7° wider when participants saw averted heads (EMM = 11.2°; SE = 0.24; CI [10.8, 11.7]) compared to direct-facing heads (EMM = 10.5°; SE = 0.24; CI [10, 11.0]) (β = -0.38; SE = 0.07, t (377.60) = -5.71; $p < 0.001$; CI [-0.50, -0.25]). However, there was no significant interaction between *Mask Condition* and *Head Orientation*. We found a significant effect of *Sex* (β = -0.59; SE = 0.24, t (134.60) = -2.46; $p = 0.015$; CI [-1.06, -0.12]), where the width of the CoDG for males was 1.1° wider (EMM = 11.4°; SE = 0.39; CI [10.68, 12.20]) than for females (EMM = 10.3°; SE = 0.27; CI [9.72, 10.80]). Social anxiety scores, nor their interaction with *Head Orientation* or *Mask Condition*, did not significantly predict the width of the CoDG.

Supplementary Table S2. Linear mixed-effects model summary including social anxiety scores as a predictor

	CoDG width (°)			
Predictors	*Estimates*	*95% CI*	*t Statistic*	*p*
**Mask Condition (MC)**	**-0.18**	**-0.31 – -0.05**	**-2.75**	**0.006**
Social Anxiety (SIAS)	-0.23	-0.67 – 0.20	-1.05	0.296
**Head Orientation (HO)**	**-0.38**	**-0.51 – -0.25**	**-5.71**	**<0.001**
Age	0.03	-0.01 – 0.06	1.50	0.135
**Sex**	**-0.59**	**-1.07 – -0.12**	**-2.46**	**0.014**
MC * SIAS	-0.12	-0.25 – 0.01	-1.79	0.074
MC * HO	-0.02	-0.15 – 0.11	-0.32	0.750
SIAS * HO	0.04	-0.09 – 0.18	0.65	0.518
MC * SIAS * HO	-0.06	-0.20 – 0.07	-0.97	0.335
**Random Effects**				
σ^2	2.21			
$\tau_{00\ PID}$	6.09			
ICC	0.73			
N_{PID}	143*			
Observations	525*			
Marginal R^2 / Conditional R^2	0.093 / 0.759			

**The number of observations and participant N are different from those reported in the main analyses, as SIAS questionnaire data for n=14 participants were removed due to a lack of engagement with the questionnaire (e.g. responding with the same response option repeatedly, irrespective of the question).*

Bolded font indicates p values less than 0.05.
